# OTULIN's influence on neuroinflammation and pain modulation in trigeminal neuralgia

**DOI:** 10.1111/cns.70006

**Published:** 2024-08-22

**Authors:** Haiyang Wang, Heng Wang, Wenhao Zheng, Ding Wang, Chenglong Sun, Jun Dong, Wenhua Yu, Quan Du

**Affiliations:** ^1^ Department of Neurosurgery, Affiliated Hangzhou First People's Hospital, School of Medicine Westlake University Hangzhou China; ^2^ Department of Neurosurgery The Second Affiliated Hospital of Soochow University Suzhou China

**Keywords:** autophagy, inflammatory markers, IONL model, neuropathic pain, NLRP3 inflammasome, OTULIN, treatment strategies, trigeminal neuralgia

## Abstract

**Introduction:**

Trigeminal neuralgia (TN), marked by chronic pain from neural damage, is closely associated with inflammation. The role of OTULIN, a key regulator in inflammation and autophagy, is not fully understood in TN. The regulatory mechanism of OTULIN, a key protein involved in modulating inflammatory responses and autophagy processes, remains incompletely elucidated, particularly in the context of TN and neuroinflammation.

**Methods:**

An infraorbital nerve ligation‐induced rat model of TN was used. OTULIN's expression was modulated using adenovirus vectors and short hairpin RNA. The impact on pain and inflammatory responses was assessed via quantitative real‐time polymerase chain reaction, western blot, immunofluorescence, and transcriptomic analysis.

**Results:**

Enhanced OTULIN expression significantly increased head withdrawal thresholds and reduced pain sensitivity and neuroinflammatory markers in the model. Conversely, silencing OTULIN exacerbated pain and inflammation. Transcriptomic data revealed OTULINs influence on both inflammatory and autophagy pathways, specifically in suppressing NLR family pyrin domain containing 3 (NLRP3) inflammasome and promoting autophagy. In vitro experiments demonstrated OTULIN's inhibition of inflammatory markers in microglia and neurons.

**Conclusion:**

OTULIN is crucial in modulating TN, reducing neuropathic pain and neuroinflammation by activating the autophagy pathway and inhibiting the NLRP3 inflammasome.

## Graphical abstract

Our study reveals that OTULIN significantly mitigates neuropathic pain and neuroinflammation in trigeminal neuralgia by activating autophagy and inhibiting the NLR family pyrin domain containing 3 inflammasome, highlighting its potential as a therapeutic target.

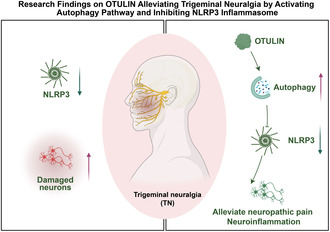

## 1 INTRODUCTION

Neuropathic pain, arising from nervous system damage, is a chronic pain state severely affecting patients' quality of life. Among its forms, trigeminal neuralgia (TN) stands out as an exceptionally agonizing condition, markedly impairing affected individuals.^1^, ^2^, ^3^, ^4^ Characterized by sudden, intense, and brief episodes of facial pain triggered by minimal facial stimuli, TN episodes resemble electric shocks, lasting anywhere from a few seconds to several minutes.^5^, ^6^, ^7^ Epidemiological findings suggest that TN patients are more susceptible to anxiety, depression, and a heightened risk of suicide.^8^, ^9^ While the understanding of TN's pathogenesis has deepened—encompassing abnormal neuronal discharge post‐nerve injury, central nervous system pathological alterations, and inflammatory response participation—the precise mechanisms remain elusive, with some attributing TN primarily to demyelination from neurovascular compression.^10^, ^11^ However, the exact pathogenic mechanisms are still not fully understood,^5^, ^12^ making elucidating genes and mechanisms that regulate TN progression crucial for developing potential treatment strategies.

Recent studies have underscored inflammation's critical role in the progression of neuropathic pain, with neuroinflammation particularly implicated in TN.^13^ The release of inflammatory mediators not only sensitizes the damaged nerve area but may also aggravates nerve injury by enhancing neuroinflammatory responses. This involves increased inflammatory mediators and infiltration by inflammatory cells, leading to abnormal signal transduction and persistent facial pain.^14^ Common inflammatory mediators associated with TN, including interleukin 1 beta (IL‐1β), TNF‐α, and IL‐6, are significant neuroinflammation markers whose elevated expression could promote neuroinflammation and neuropathic pain progression.^15^, ^16^, ^17^ Microglial cells, a type of inflammatory cell, are closely associated with neuroinflammation. Their activation, another neuroinflammation indicator, exacerbates neuropathic pain by facilitating communication with neurons,^18^, ^19^ highlighting the importance of studying neuroinflammation for a thorough understanding of TN's underlying mechanisms.

The NLR family pyrin domain containing 3 (NLRP3) inflammasome plays a crucial role in regulating immune and inflammatory signaling pathways.^20^, ^21^ It is composed of the NLRP3 scaffold, the apoptosis‐associated speck‐like protein (ASC) adaptor, and the caspase‐1 effector.^22^, ^23^, ^24^ Upon activation, NLRP3 recruits and activates caspase‐1, facilitating the maturation and secretion of IL‐1β and IL‐18.^25^, ^26^, ^27^ Increasing evidence suggests that hyperactivation of the NLRP3 inflammasome is associated with a variety of neurological diseases, including neuropathic pain,^28^ infections, neurotrauma,^29^ and Alzheimer's disease.^30^, ^31^ IL‐1β, produced by NLRP3 inflammasome activation, intensifies the communication between neurons and neuroglia, leading to neural sensitization and enhanced pain.^32^ Despite the broad implications of the NLRP3 inflammasome, its role in the context of TN has yet to be thoroughly explored.

Autophagy, a cellular self‐degradation and recycling process, plays a crucial role in clearing intracellular waste, maintaining cellular environmental stability, and providing energy for cells.^32^, ^33^, ^34^ It is essential for regulating immune inflammation and maintaining neurological homeostasis.^35^ Autophagy is involved in the process of nerve damage and the formation of neuropathic pain.^36^, ^37^ Studies on animal models have shown significant changes in the autophagic activity of damaged nerves, spinal cord, and brain.^36^ The upregulation of autophagy is associated with neuropathic pain,^38^ yet its role in TN remains unclear.

OTULIN, also known as Gumby/FAM105b, is a member of the deubiquitinase family that plays a key role in regulating abnormal immune responses and maintaining tissue homeostasis.^39^ As a critical regulator of inflammatory responses and autophagy processes, OTULIN has shown significant importance in various inflammation‐related diseases. Overexpression of OTULIN in a rat model of cerebral ischemia/reperfusion inhibits the activation of the NF‐κB signaling pathway, thereby improving microglial activation and neuroinflammation.^40^ Maintaining a balance of linear ubiquitination is beneficial for physiological autophagy, an essential cellular survival mechanism involved in the clearance of aged or damaged proteins and organelles and in controlling inflammation. Appropriate ubiquitination of ATG13 can initiate autophagy.^41^, ^42^ However, excessive ubiquitination of ATG13 can hinder the maturation of autophagosomes, and knocking down OTULIN expression inhibits the maturation of autophagosomes,^43^ indicating OTULIN's crucial role in autophagosome formation. Nevertheless, the function of OTULIN in neuropathic pain has not been reported. The specific role and mechanism of action of OTULIN as a pivotal protein regulating inflammation and autophagy in the TN model have not been extensively investigated.

We anticipate that this research will elucidate OTULIN's regulatory role in TN and neuroinflammation, revealing how it alleviates neuropathic pain and neuroinflammation by activating autophagy processes and inhibiting NLRP3 inflammasome activity. The results of this study are expected to provide new insights into the pathological mechanisms of TN and offer a scientific basis for developing new therapeutic strategies holding significant scientific and clinical importance.

## 2 MATERIALS AND METHODS

### 2.1 Transcriptome sequencing and data processing

Next‐generation sequencing technology was employed to perform transcriptome sequencing on the trigeminal ganglia of model rats. Total RNA was extracted from tissue samples using the TruSeq RNA Sample Preparation Kit (Illumina), and cDNA libraries were constructed. High‐throughput sequencing was conducted on the Illumina HiSeq platform to obtain raw sequencing data. The raw paired‐end reads' quality was assessed using FastQC software v0.11.8 (http://www.bioinformatics.babraham.ac.uk). Pre‐processing of the raw data was carried out using Cutadapt software 1.18 (http://www.bioinformatics.babraham.ac.uk) to remove Illumina sequencing adapters and poly(A) tail sequences. Reads with over 5% N content were filtered out using a Perl script. Reads with a base quality of over 20 and comprising 70% of the bases were extracted using FASTX Toolkit software 0.0.13 (http://hannonlab.cshl.edu/fastx_toolkit/). BBMap software (https://sourceforge.net/projects/bbmap/) was employed to repair paired‐end sequences. Subsequently, high‐quality filtered reads were aligned to the rat reference genome using HISAT2 software version 0.7.12.^44^, ^45^

### 2.2 Differential expression gene analysis (DEGs)

Gene expression quantification and differential analysis were conducted using the StringTie and Ballgown suite. A threshold of |log2(fold change)| >1 and *p* < 0.05 was set for filtering differentially expressed genes. The results of DEGs were visualized using the Heatmap tool to identify genes with significant expression changes following OTULIN upregulation.

### 2.3 Gene ontology (GO) and pathway (KEGG) enrichment analysis

The ClusterProfiler R package was utilized for gene ontology (GO) function and KEGG pathway enrichment analysis of DEGs to explore the role of OTULIN upregulation in biological processes such as inflammation and autophagy. A *p* < 0.05 was established as the significance level for enrichment, and results were visualized using the Enrichment Map plugin to identify the main biological signaling pathways affected by OTULIN upregulation.

### 2.4 Gene set enrichment analysis (GSEA)

Gene set enrichment analysis (GSEA) software was further used to analyze the impact of OTULIN upregulation on specific functional gene sets. Autophagy and inflammation‐related gene sets were selected as reference gene sets for the GSEA, assessing the overall effect of OTULIN upregulation on the expression of these gene sets. A false discovery rate (FDR) <0.25 and a normalized enrichment score (NES) >1 were set as thresholds for significant enrichment.

### 2.5 Establishment of the animal experimental model

In this study, to thoroughly investigate the mechanisms behind TN and its associated neuroinflammation induced by infraorbital nerve ligation (IONL), Sprague–Dawley rats weighing between 200 and 250 g were selected as experimental subjects. All rats were housed in a specific pathogen‐free (SPF) environment to ensure the accuracy and reliability of the research. The environmental temperature was strictly controlled at 22 ± 1°C, with a 12‐h light/dark cycle maintained to simulate a natural ecological setting and ensure the physiological rhythm and health of the animals. To minimize stress responses and acclimate the rats to the experimental conditions, a 3‐day environmental acclimatization period was provided before the onset of the experiments, during which the rats had free access to food and water. The animal experimental protocol for this study was approved by the Ethics Committee of Zhejiang University School of Medicine, approval number “ZJCLA‐IACUC‐20010543,” and was conducted strictly in accordance with the ethical guidelines issued by the International Association for the Study of Pain (IASP) to ensure the ethical and scientific integrity of the experiment.

### 2.6 Precise establishment of the rat model of trigeminal neuralgia

To establish the rat model of TN, the study employed the IONL technique.^46^ Initially, rats were anesthetized through intraperitoneal injection of 4% sodium pentobarbital (dose: 50 mg/kg) to ensure they remained pain‐free during the surgical procedure. Subsequently, an incision was made between the mucosa and the first molar of the rat's left cheek to expose the infraorbital nerve. This step required precise handling to avoid unnecessary damage to surrounding tissues. Once the nerve was exposed, it was ligated at both proximal and distal ends with two 5–0 absorbable sutures (chromic catgut) to simulate the pathological state of TN by reducing the nerve's diameter while carefully avoiding complete obstruction of subcutaneous blood circulation to maintain tissue physiological function. After the surgery, the mucosal tissue of the cheek was sutured to promote wound healing. Rats in the sham surgery group underwent the same surgical procedure but without nerve ligation to assess the impact of the ligation surgery itself on rat behavior and physiology.

### 2.7 von Frey test

In this study, the von Frey test was employed to assess changes in the mechanical sensitivity of rats' facial areas, particularly before and after the IONL procedure, to determine alterations in facial pain sensitivity.^47^ Initially, rats were placed in a metal mesh cage to allow for an adaptation period of 0.5–1 h before undergoing the facial stimulation test, a crucial step to minimize the potential impact of environmental factors on the test outcomes. At the start of the test, researchers gently applied von Frey filaments to the whisker pad skin of rats, gradually increasing the stimulus intensity until a withdrawal response was observed. To ensure the accuracy of the test, a 5‐s interval was maintained between each stimulus, and each intensity level was repeated five times. The head withdrawal threshold (HWT) was defined as the lowest mechanical stimulus intensity that elicited a withdrawal response in at least three out of five stimuli. Furthermore, to guarantee the reliability of the data, a cut‐off value of 100 g was established to prevent additional harm or discomfort that might be caused by excessive stimuli.

### 2.8 Face‐grooming test

The face‐grooming test was utilized in this research to evaluate the facial scratching and grooming behavior of rats under specific conditions. Prior to the test, rats were first placed in a wire mesh enclosure, allowing them free movement within the new environment for 0.5–1 h to achieve adaptation. This adaptation period is vital for reducing the impact of external environmental changes on rat behavior, ensuring the accuracy and reliability of the test results. During the test, researchers closely monitored and accurately recorded the frequency of facial scratching and grooming behaviors exhibited by the rats over a continuous 10‐min period. These behaviors are considered indicators of pain or discomfort; thus, by quantifying the frequency of these behaviors, an indirect assessment of the rats' facial sensitivity and potential changes in pain perception can be made. To ensure the consistency and accuracy of data collection, all observations were conducted by specially trained observers in a quiet environment to avoid sound disturbances that could interfere with the rats' natural behavior.

### 2.9 Viral and pharmacological administration via trigeminal ganglion

In this study, adeno‐associated virus overexpression vectors (AAV‐OTULIN), short hairpin RNA (shOTULIN) knockdown vectors targeting OTULIN, and their respective negative controls were custom‐constructed by GenePharma. The short hairpin RNA (shRNA) sequences were as follows: shOTULIN‐1: CTGAGGATGATCGGCACTATA; shOTULIN‐2: CTCAGAAATTCACTTCCATAA; and shOTULIN‐3: GAAGACTAGCTGTTGATAATG. A multi‐shRNA strategy was employed, involving co‐injection of AAV‐shRNAs targeting distinct sites to mitigate off‐target effects.^48^ Additionally, NLRP3 inflammasome activators and the autophagy inhibitor 3‐methyladenine (3‐MA) were procured from MedChemExpress for subsequent pharmacological investigations. For the administration experiments, rats were deeply anesthetized with 4% sodium pentobarbital to ensure a painless procedure throughout the surgery. Rats were positioned supine on the surgical table, and through precise anatomical localization, the posterior side of the zygomatic arch of the maxilla was identified. Subsequently, a 25‐gauge sterile insulated needle was inserted from the inner side of the infraorbital foramen (1–2 mm) and advanced approximately 2.2 cm along the infraorbital canal, passing through the round foramen, ultimately positioning inside the trigeminal ganglion.^49^

Upon accurate placement of the needle tip within the trigeminal ganglion, viral vectors (concentration of 10^9^ TU) and pharmacological agents (QS‐21 at 5 μg/100 g body weight; 3‐MA at 0.5 mg/100 g body weight) were slowly injected. This procedure required utmost precision to ensure direct effects on the trigeminal ganglion, thereby maximally simulating disease states and evaluating therapeutic outcomes.

### 2.10 Primary culture of rat trigeminal ganglion neurons and microglia, viral transfection, and pharmacological treatment

This research details the process of primary culture of neurons and microglia from rat TGs, followed by subsequent viral transfection and pharmacological treatment. Initially, rats were euthanized via cervical dislocation under deep anesthesia to ensure humane treatment throughout the experiment. Trigeminal ganglions were aseptically excised, repeatedly rinsed with ice‐cold balanced salt solution, minced, and digested in papain solution (2 mg/mL) for 0.5 h. Following digestion, the reaction was terminated with DMEM/F12 medium containing 10% FBS, and cells were gently triturated and seeded onto dishes pre‐coated with 0.01% poly‐L‐lysine (Sigma). To foster neuronal growth, the medium was subsequently replaced with serum‐free neurobasal medium (Thermo Fisher) supplemented with B27 and glutamate. On the third day of culture, cytosine arabinoside (Ara‐C) was added to inhibit the growth of non‐neuronal cells.

The primary culture of microglia followed tissue procurement and digestion steps similar to those of neurons. Microglia were isolated using the shaking method^50^, ^51^ and cultured in DMEM/F12 medium supplemented with 10% FBS and 10 ng/mL M‐CSF until stratified growth was observed. The partially suspended growth characteristic of microglia was exploited for purification through shaking, and the collected cells were further cultured in 6‐well plates.

When cells reached 70%–80% confluence, viral transfection was performed using Lipofectamine 3000 (Invitrogen), transfecting AAV‐OTULIN, shOTULIN, and their respective negative control vectors. Forty‐eight hours post‐transfection, cells were treated with QS‐21 (2 μg/mL) and 3‐MA (5 mM) to assess the effects of pharmacological interventions on cellular responses.

### 2.11 qRT‐PCR

In this study, the expression levels of specific genes within the TGs were analyzed using quantitative real‐time polymerase chain reaction (qRT‐PCR). Total RNA was extracted from the TG samples using the TRIzol reagent (Invitrogen) and reverse transcribed into cDNA using a reverse transcription kit (Thermo Fisher), following the manufacturer's guidelines. For the qPCR analysis, SYBR Green PCR Master Mix (Vazyme Biotech) was utilized, which is crucial for achieving high sensitivity and specificity in DNA amplification. The qPCR amplification protocol included an initial denaturation step at 95°C for 30 s, followed by 40 cycles of denaturation at 95°C for 5 s and annealing/extension at 60°C for 45 s, concluding with a final denaturation step at 95°C for 15 s to ensure complete denaturation. GAPDH was employed as an endogenous control gene for normalization to correct for variations between samples.

After PCR amplification, the specificity of the amplification products was confirmed through melt curve analysis, ensuring the accuracy of the experimental results. Data analysis was conducted using the 2^−ΔΔCt^ method, where the cycle threshold (Ct) values of the target genes were normalized and quantitatively analyzed relative to the Ct values of GAPDH, calculating the relative changes in gene expression. Detailed information on the primer sequences is provided in Table 1.

**TABLE 1:** Primer sequences for qRT‐PCR analysis of OTULIN and GAPDH.

Name	Forward (5′‐3′)	Reverse (5′‐3′)
OTULIN	GGACACATCAAATGACCCAGGACAG	CGCACAGCATAGGCAAGAAGGAAC
GAPDH	AAGTTCAACGGCACAGTCAAGG	GACATACTCAGCACCAGCATCAC

### 2.12 Western blot analysis

This research employed western blotting to intricately analyze the expression of specific proteins in the TGs and cells. Samples were homogenized in RIPA lysis buffer (Beyotime) in the presence of protease and phosphatase inhibitors to ensure efficient protein extraction. The extracted proteins were then quantified using a BCA protein assay kit (Beyotime), ensuring consistent protein loading across samples for subsequent experiments.

For the electrophoresis process, 30 μg of protein from each sample was separated by SDS‐PAGE and then transferred to PVDF membranes (millipore). Prior to specific antibody detection, membranes were pre‐blocked with a 5% milk solution to reduce background interference. The membranes were then incubated with a series of antibodies, including OTULIN, calcitonin gene‐related peptide (CGRP), post‐synaptic density protein 95 (PSD95), ionized calcium‐binding adapter molecule 1 (IBA‐1), IL‐1β, tumor necrosis factor alpha (TNFα), NLRP3, ASC, caspase‐1, microtubule‐associated proteins 1A/1B light chain 3 (LC3), and GAPDH as the internal reference (all antibodies supplied by Abcam, used at a concentration of 1:1000, except GAPDH at 1:5000). After incubation with primary antibodies, membranes were treated with diluted secondary antibodies (Beyotime, 1:10000 concentration) at room temperature for 1 h to achieve signal labeling through secondary antibody binding. Enhanced chemiluminescence (ECL) technology was used for visualization of the labeled proteins, and the intensity of protein bands was quantitatively analyzed using ImageJ software (NIH), thereby accurately assessing the expression levels of each target protein in the samples.

### 2.13 Immunofluorescence staining of trigeminal ganglia, neurons, and microglia

In this study, immunofluorescence staining was employed to perform detailed protein expression localization analysis in trigeminal ganglia, as well as in neurons and microglia. Initially, trigeminal ganglia samples were fixed overnight at 4°C using 4% paraformaldehyde to preserve tissue integrity. Following fixation, the samples were subjected to cryoprotection in 30% sucrose solution for at least 48 h to prevent ice crystal damage during subsequent cryosectioning. The samples were then embedded in Tissue‐Tek and sectioned at 10 microns thickness under −20°C conditions.

For cell samples, neurons and microglia seeded on confocal dishes were fixed using 4% paraformaldehyde. After fixation, the samples were treated with 0.25% Triton X‐100 (Solarbio) to increase membrane permeability, facilitating antibody penetration. Subsequently, samples were blocked with 5% bovine serum albumin (BSA) at room temperature for 1 h to reduce nonspecific background staining. Incubation with primary antibodies was conducted overnight at 4°C, using primary antibodies against OTULIN, IBA‐1, NLRP3, LC3, and NeuN (all from Abcam, diluted 1:200). Following primary antibody incubation, samples were treated with secondary antibodies (Servicebio, diluted 1:300) carrying different fluorescent labels such as Cy3, Alexa Fluor 488, Alexa Fluor 594, or Cy5 for specific antibody fluorescence marking, at room temperature for 1 h. Nuclei were stained with 4′,6‐diamidino‐2‐phenylindole (DAPI; Beyotime) for 10 min to visualize cell nuclei under a fluorescence microscope. Stained sections and cells were observed with a fluorescence microscope, and the images obtained were processed and analyzed using ImageJ software.

### 2.14 Statistical analysis

The statistical and visualization analysis in this study was conducted using R software (version 4.0 or higher) and GraphPad Prism software (version 8.0.2) for data processing and presentation. All data were assessed for normality of distribution using the Shapiro–Wilk test. Parametric tests were applied to normally distributed data, while non‐parametric tests were used for skewed data. All statistical analyses were presented as mean ± standard deviation and organized and graphed using GraphPad Prism. Heatmaps of DEGs were generated using the pheatmap package in R software, while GSEA results were displayed using the default plotting functions of GSEA software. All in vitro experiments were repeated at least three times, and in vivo experiments included a minimum of five rats per group to ensure the reliability and statistical power of the experiments. The assessment of significant differences between experimental groups was based on the data distribution and the number of comparison groups, employing one‐way or two‐way ANOVA and Student's *t*‐test as applicable. All statistical analyses were conducted with a significance level set at *p* < 0.05, aimed at thoroughly analyzing the interactions and differences between experimental variables.

## 3 RESULTS

### 3.1 Upregulation of OTULIN expression in rat models of trigeminal neuralgia and its correlation with neuropathic pain

**FIGURE 1:**
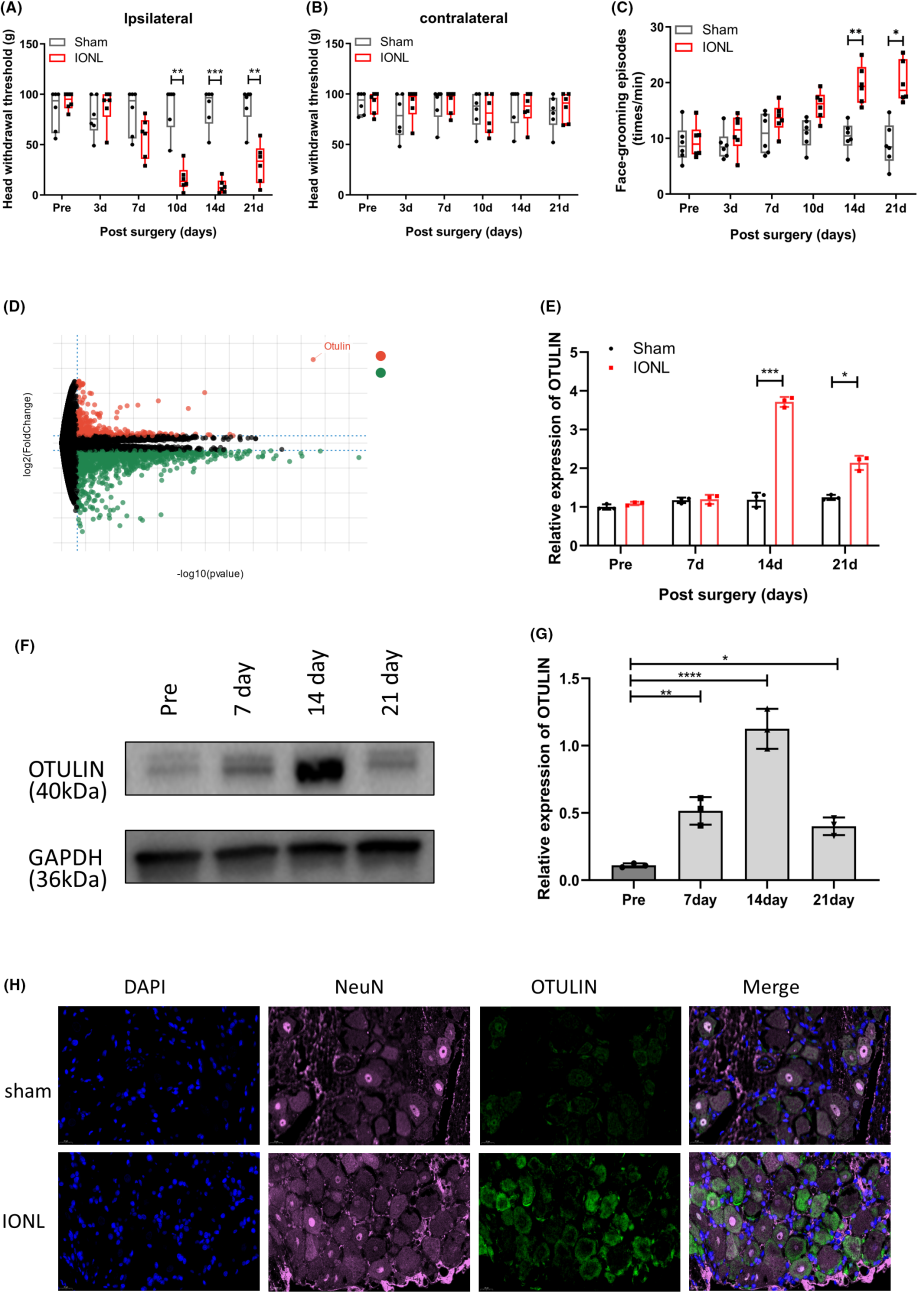
Correlation analysis of OTULIN expression and pain behavior in the IONL‐induced trigeminal neuralgia model. (A) The von Frey test was utilized to assess the ipsilateral head withdrawal threshold in IONL rats, comparing data at post‐surgery Days 10, 14, and 21 to quantify mechanical allodynia. (B) The von Frey test was also applied to assess the contralateral head withdrawal threshold, confirming the specificity of the surgical side effect. (C) The face‐grooming behavior test evaluated the number of face‐grooming episodes in IONL rats at post‐surgery Days 14 and 21, serving as an indicator of spontaneous pain behavior. (D) mRNA sequencing analysis highlighted differentially expressed genes in the trigeminal ganglia of IONL and sham‐operated rats, with a focus on OTULIN expression changes. (E) qRT‐PCR was employed to quantitatively assess OTULIN mRNA levels, further validating the mRNA sequencing results. (F) The detection of the OTULIN protein expression level via immunoblot experiment. (G) qRT‐PCR evaluated OTULIN mRNA expression levels. (H) Immunofluorescence staining, employing dual labeling of OTULIN and the neuronal marker NeuN, illustrated OTULIN expression and distribution in the trigeminal ganglia. Nuclei were stained blue with 4′,6‐diamidino‐2‐phenylindole (DAPI), providing background information on cell localization and morphology; NeuN was marked with deep purple fluorescence to identify neurons; OTULIN was marked with green fluorescence to indicate its expression in neurons. The sample size per group was *n* = 5. Data were analyzed using two‐way ANOVA, with significance levels marked as *p < 0.05, **p < 0.01, ***p < 0.001, ****p < 0.0001.

Compared with sham‐operated rats, the IONL rats exhibited a significant reduction in head withdrawal thresholds on the ipsilateral side on Days 10, 14, and 21 post‐surgery, with no significant changes observed on the contralateral side (Figure 1A,B). Additionally, face‐grooming behavior tests indicated that, on Days 14 and 21 post‐surgery, IONL rats engaged in more frequent face‐grooming behaviors than sham‐operated IONL rats (Figure 1C), suggesting the successful establishment of a TN model in rats via IONL.

To investigate the etiology of neuropathic pain, we collected ipsilateral trigeminal ganglions on Day 14 post‐IONL and analyzed the gene expression profiles via mRNA sequencing. The results revealed a significant increase in OTULIN expression in the trigeminal ganglions of IONL rats (Figure 1D). Subsequent qRT‐PCR analysis of OTULIN expression on Days 7, 14, and 21 post‐IONL (Figure 1E) and western blot results indicated a marked upregulation of OTULIN on Day 14, which remained elevated on Day 21, aligning with the timeline of mechanical allodynia development (Figure 1F,G). Moreover, immunofluorescence staining of the trigeminal ganglions post‐IONL showed a significant increase in OTULIN expression within neurons compared with the control group not subjected to IONL (Figure 1H). Collectively, this study revealed the upregulation of OTULIN in an IONL‐induced neuropathic pain model and demonstrated a close association between its expression changes and the occurrence of pain behaviors.

### 3.2 OTULIN modulates neuropathic pain and neuroinflammatory markers to alleviate infraorbital nerve ligation (IONL)‐induced trigeminal neuralgia

Employing an AAV vector to upregulate OTULIN expression (AAV‐oeOTULIN) and comparing it with a negative control vector (oeNC), we conducted precise genetic manipulation in a rat model, targeting injections into the trigeminal ganglion.

**FIGURE 2:**
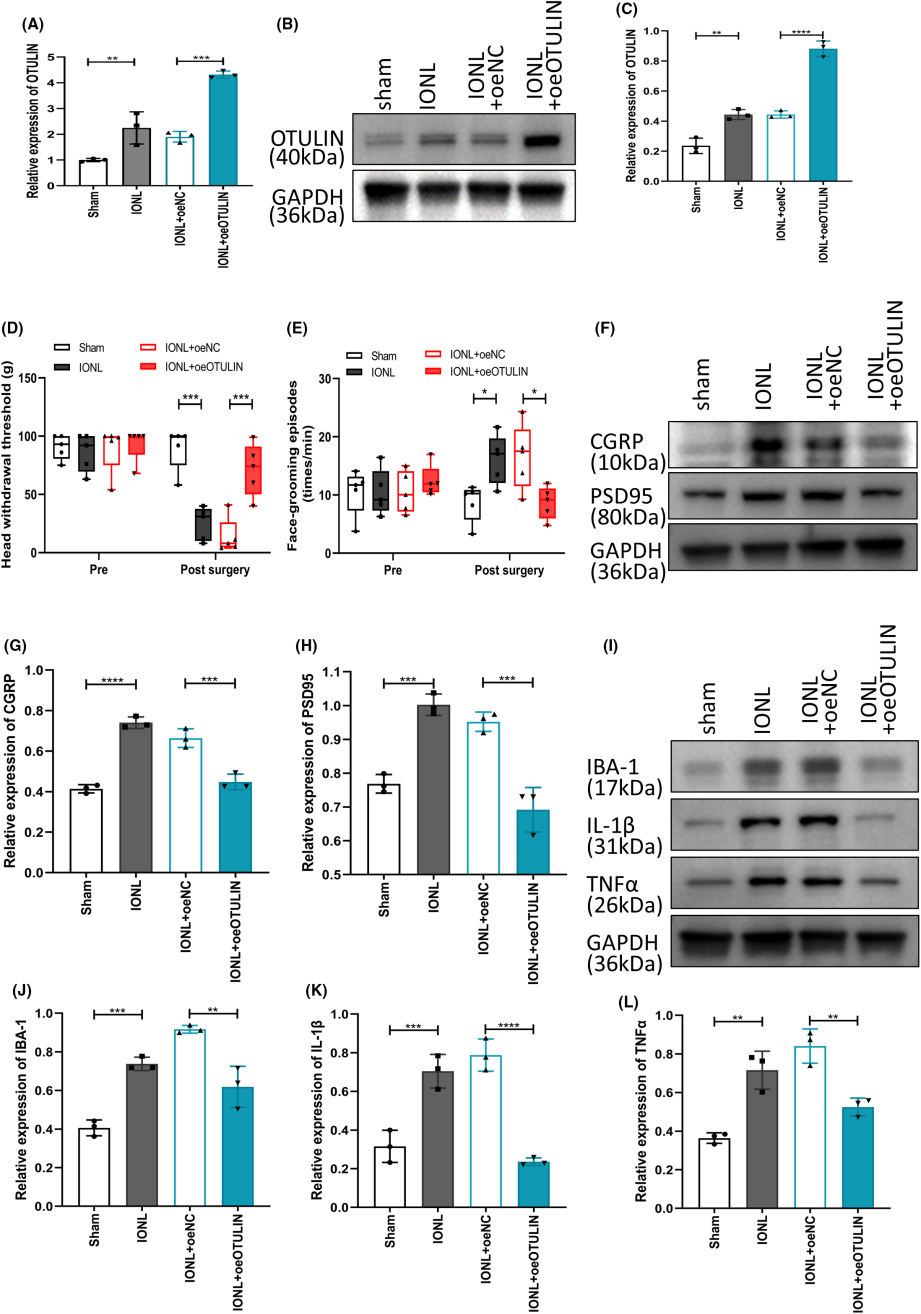
Analysis of OTULIN overexpression in IONL‐induced trigeminal neuralgia and neuroinflammation. (A) Real‐time quantitative PCR technology assessed OTULIN expression levels in rat trigeminal ganglia (TG), comparing the IONL+oeOTULIN group with the IONL+oeNC and sham surgery groups to quantitatively analyze the effects of OTULIN overexpression. (B, C) Immunoblotting further verified OTULIN protein expression in the TG, comparing differences among experimental groups and reinforcing the reliability of qRT‐PCR results. (D) The von Frey test measured the head withdrawal threshold of rats in each group, reflecting changes in mechanical pain sensitivity and directly showing the impact of OTULIN overexpression on pain perception. (E) The face‐grooming test recorded the number of face‐grooming behaviors in each group, serving as another indicator to assess the regulatory effects of OTULIN overexpression on spontaneous pain behavior. (F–L) Immunoblotting analyzed expression levels of neuropathic pain markers (CGRP and PSD95) and neuroinflammation markers (IBA‐1, IL‐1β, and TNFα) in the TG, comprehensively evaluating the impact of OTULIN overexpression on neuropathic pain and neuroinflammation. The sample size per group was *n* = 5. Statistical significance was determined using one‐way ANOVA, with significance levels marked as *p < 0.05, **p < 0.01, ***p < 0.001, ****p < 0.0001, ensuring the scientific rigor and statistical validity of the experimental results.

Through qRT‐PCR (Figure 2A) and western blot analyses (Figure 2B,C), this study successfully confirmed significant upregulation of OTULIN in the IONL model rats. It was observed that, compared with the sham surgery group, overexpression of OTULIN significantly increased the head withdrawal threshold (Figure 2D) and decreased the frequency of face grooming (Figure 2E) in IONL rats, both critical indicators for assessing pain perception and spontaneous pain behaviors. Furthermore, immunoblotting analysis of neuropathic pain markers (CGRP and PSD95)^52^, ^53^, ^54^ and neuroinflammatory markers (IBA‐1, IL‐1β, and TNFα) revealed that the expression levels of these markers in the IONL group TG were higher than those in the sham surgery group. In contrast, in the oeOTULIN IONL‐TG, the expression levels of these markers were significantly lower than those in the oeNC IONL‐TG (Figure 2F–H). Similarly, the expression of IBA‐1, IL‐1β, and TNFα was upregulated in the IONL‐TG compared with the sham‐TG. The expression levels of IBA‐1, IL‐1β, and TNFα in the IONL‐TG with oeOTULIN were reduced compared to those with oeNC (Figure 2I–L). These findings confirm that overexpression of OTULIN effectively reduces the levels of these pain‐sensitive and inflammation‐related proteins, thereby revealing a significant mitigating effect of OTULIN on IONL‐induced TN and neuroinflammation.

### 3.3 Analysis of OTULIN knockdown's role in exacerbating IONL‐induced trigeminal neuralgia and neuroinflammation

**FIGURE 3:**
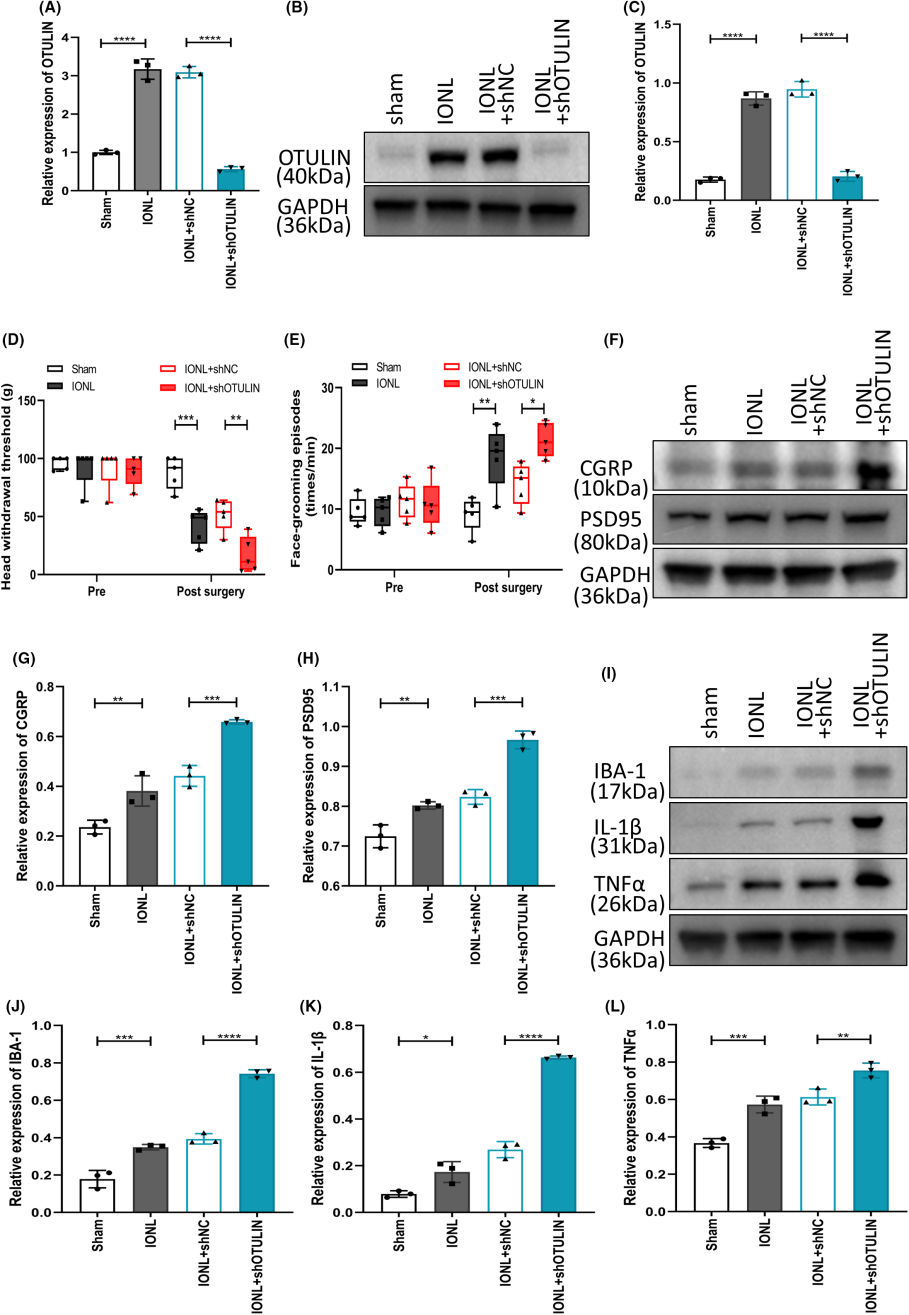
Impact of OTULIN Silencing on IONL‐Induced Trigeminal Neuralgia and Neuroinflammation. (A) Real‐time quantitative PCR (qRT‐PCR) analysis evaluated OTULIN expression levels in rat trigeminal ganglia (TG), with experimental groups including sham surgery, IONL, IONL+shNC, and IONL+shOTULIN. (B, C) Immunoblotting (western blot) quantified OTULIN protein expression in the TG. (D) The von Frey experiment measured head withdrawal thresholds for each group, assessing the degree of mechanical pain allodynia. (E) The face‐grooming experiment recorded face‐grooming counts for each group, serving as an indicator of spontaneous pain behavior. (F–L) Immunoblotting further evaluated the expression levels of neuropathic pain markers (CGRP and PSD95) and neuroinflammation markers (IBA‐1, IL‐1β, and TNFα) in the TG, revealing the effects of OTULIN silencing on these markers. The sample size per group was *n* = 5. Statistical analysis was conducted using one‐way ANOVA, with significance levels marked as *p < 0.05, **p < 0.01, ***p < 0.001, ****p < 0.0001, visually presenting significant differences between experimental groups.

In this experiment, shOTULIN and shNC were injected into the TG of rats on the third day post‐IONL, followed by an assessment of neuropathic pain behavior on the 14th day post‐surgery and collection of TG samples for molecular biological analysis. Through qRT‐PCR (Figure 3A) and immunoblotting experiments (Figure 3B,C), we confirmed a significant reduction in OTULIN expression levels in IONL‐TGs carrying shOTULIN, verifying effective gene knockdown. Results from the von Frey test and face‐grooming test indicated that shOTULIN‐treated IONL rats exhibited a decreased head withdrawal threshold (Figure 3D) and a significant increase in face‐grooming frequency (Figure 3E), suggesting exacerbated pain behavior. Furthermore, immunoblotting analysis revealed a significant upregulation in the expression of neuropathic pain markers (CGRP and PSD95) and neuroinflammatory markers (IBA‐1, IL‐1β, and TNFα) in OTULIN‐silenced IONL‐TGs (Figure 3F–L), indicating intensified neuroinflammatory responses.

These findings demonstrate that OTULIN knockdown significantly exacerbates pain behavior and neuroinflammatory responses in the IONL‐induced TN model, revealing OTULIN's potential protective role in regulating neuropathic pain and neuroinflammation.

### 3.4 OTULIN upregulation alleviates trigeminal neuralgia and neuroinflammation by activating autophagy and inhibiting inflammatory signaling pathways

**FIGURE 4:**
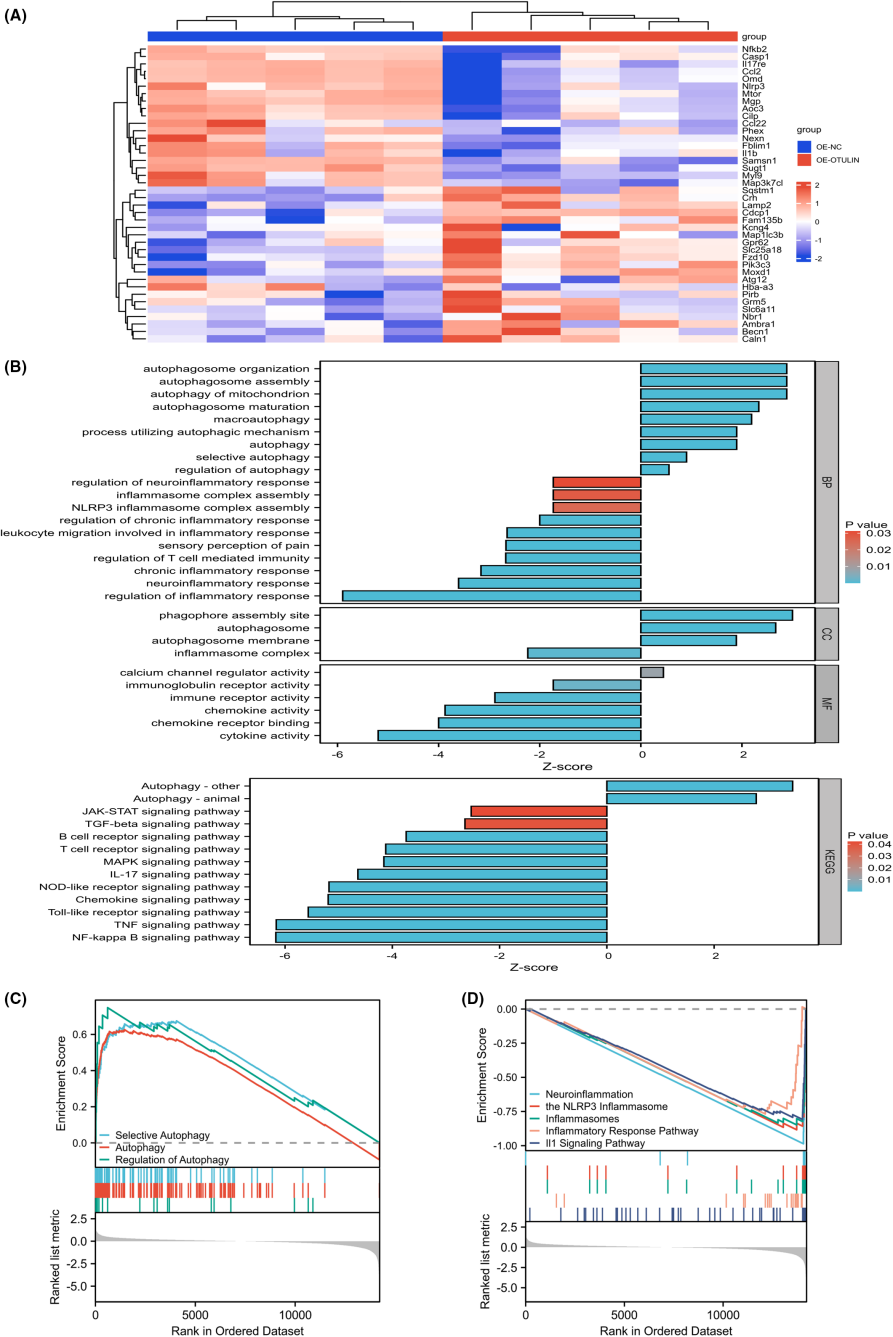
Enrichment analysis of transcriptional impact in trigeminal ganglia by OTULIN upregulation. (A) Transcriptome sequencing analyzed differentially expressed genes (DEGs) in TG‐carrying oeOTULIN or oeNC, aiming to reveal the effects of OTULIN expression changes on gene expression patterns. (B) Gene Ontology (GO) and Kyoto Encyclopedia of Genes and Genomes (KEGG) enrichment analyses categorized DEGs to identify the main biological processes and signaling pathways affected by OTULIN upregulation in the TG. (C, D) Gene Set Enrichment Analysis (GSEA) further assessed the distribution of OTULIN‐related DEGs in autophagy and inflammation signaling pathways.

Transcriptome sequencing analysis of TG post‐transfection with oeOTULIN and oeNC revealed that the upregulation of OTULIN significantly impacts the inflammation‐related signaling pathways and autophagy‐related signaling pathways in the TG (Figure 4A). Specifically, results from Gene Ontology (GO), Kyoto Encyclopedia of Genes and Genomes (KEGG), and Gene Set Enrichment Analysis (GSEA) indicated that OTULIN upregulation substantially inhibited inflammatory signaling pathways, including neuroinflammation, NLRP3 inflammasome, NF‐κB, IL‐1, TNF, among others. Concurrently, autophagy‐related signaling pathways, such as autophagy regulation and selective autophagy, were significantly activated in the TG with OTULIN upregulation (Figure 4B–D and Figure S1). These enrichment analysis results not only reveal the complex role of OTULIN in regulating TN and neuroinflammation but also suggest that OTULIN may alleviate symptoms of TN and neuroinflammation by simultaneously activating autophagy processes and inhibiting inflammatory signaling pathways.

### 3.5 OTULIN alleviates IONL‐induced trigeminal neuralgia and neuroinflammation by inhibiting the NLRP3 inflammasome

**FIGURE 5:**
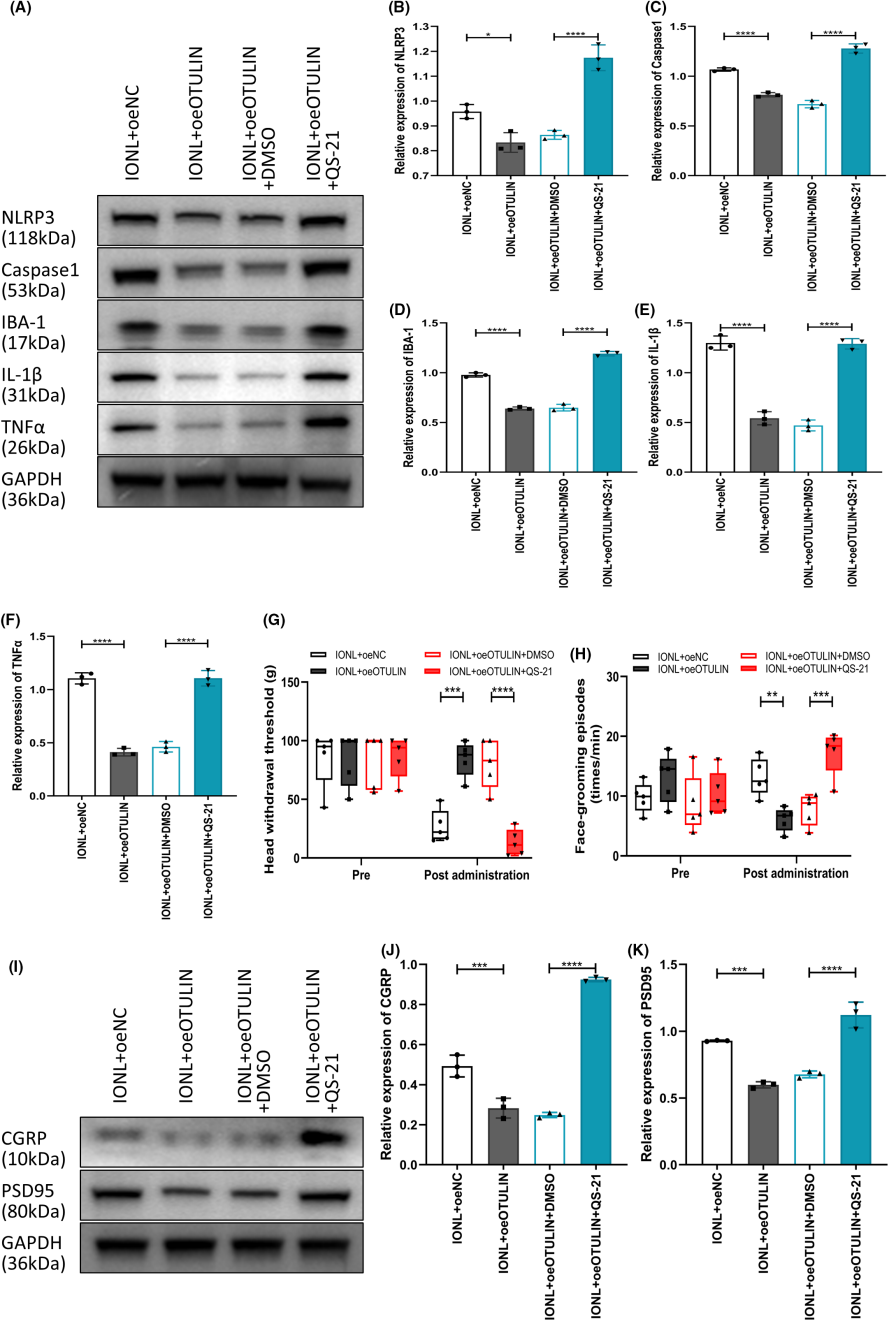
Reversal of OTULIN inhibitory effects by NLRP3 inflammasome activator intervention. (A–F) Immunoblotting assessed the expression levels of OTULIN, NLRP3 inflammasome components (NLRP3 and caspase‐1), and neuroinflammation markers (IBA‐1, IL‐1β, TNFα) in TG of different treatment groups. (G) The von Frey test quantified changes in pain sensitivity, comparing rats treated only with oeOTULIN to those also treated with oeOTULIN and QS‐21. (H) The face‐grooming behavior test recorded the number of face‐grooming episodes in different treatment groups, assessing differences in spontaneous pain behavior. (I–K) Immunoblotting further analyzed the expression of neuropathic pain markers (CGRP and PSD95) in the TG, evaluating the reversal of OTULIN inhibitory effects. The sample size per group was *n* = 5. Statistical significance was determined using one‐way ANOVA, with significance levels marked as *p < 0.05, **p < 0.01, ***p < 0.001, ****p < 0.0001.

The results indicate that the upregulation of OTULIN significantly suppressed the expression of NLRP3 and caspase‐1, core components of the NLRP3 inflammasome, essential for alleviating neuroinflammation.^55^, ^56^ Concurrently, upregulation of OTULIN led to reduced expression of neuroinflammatory markers (IBA‐1, IL‐1β, and TNFα). By injecting the NLRP3 inflammasome activator QS‐21 into TGs carrying oeOTULIN, we observed a reversal of OTULIN's inhibitory effects on neuroinflammatory markers (Figure 5A–F), further confirming that OTULIN mitigates IONL‐induced neuroinflammation by inhibiting the NLRP3 inflammasome.

Behavioral experiments revealed that upregulation of OTULIN increased the head withdrawal threshold and decreased the frequency of face grooming (Figure 5G,H), effects that were reversed upon co‐administration of QS‐21, illustrating OTULIN's analgesic and anti‐inflammatory actions through NLRP3 inflammasome inhibition. Additionally, immunoblot analysis of neuropathic pain markers (CGRP and PSD95) further validated that OTULIN's inhibitory effects could be reversed by intervention with the NLRP3 inflammasome activator (Figure 5I–K).

In summary, this study elucidates the critical regulatory role of OTULIN in mitigating IONL‐induced TN and neuroinflammation by inhibiting the NLRP3 inflammasome.

### 3.6 Mechanistic study on OTULIN's inhibition of NLRP3 inflammasome activity induced by IONL via autophagy activation

Building on previous findings, we discovered that OTULIN suppresses NLRP3 inflammasome activity by modulating the autophagy process. Enrichment analyses further elucidated the mechanism by which OTULIN may inhibit the NLRP3 inflammasome through the activation of autophagy levels.

**FIGURE 6:**
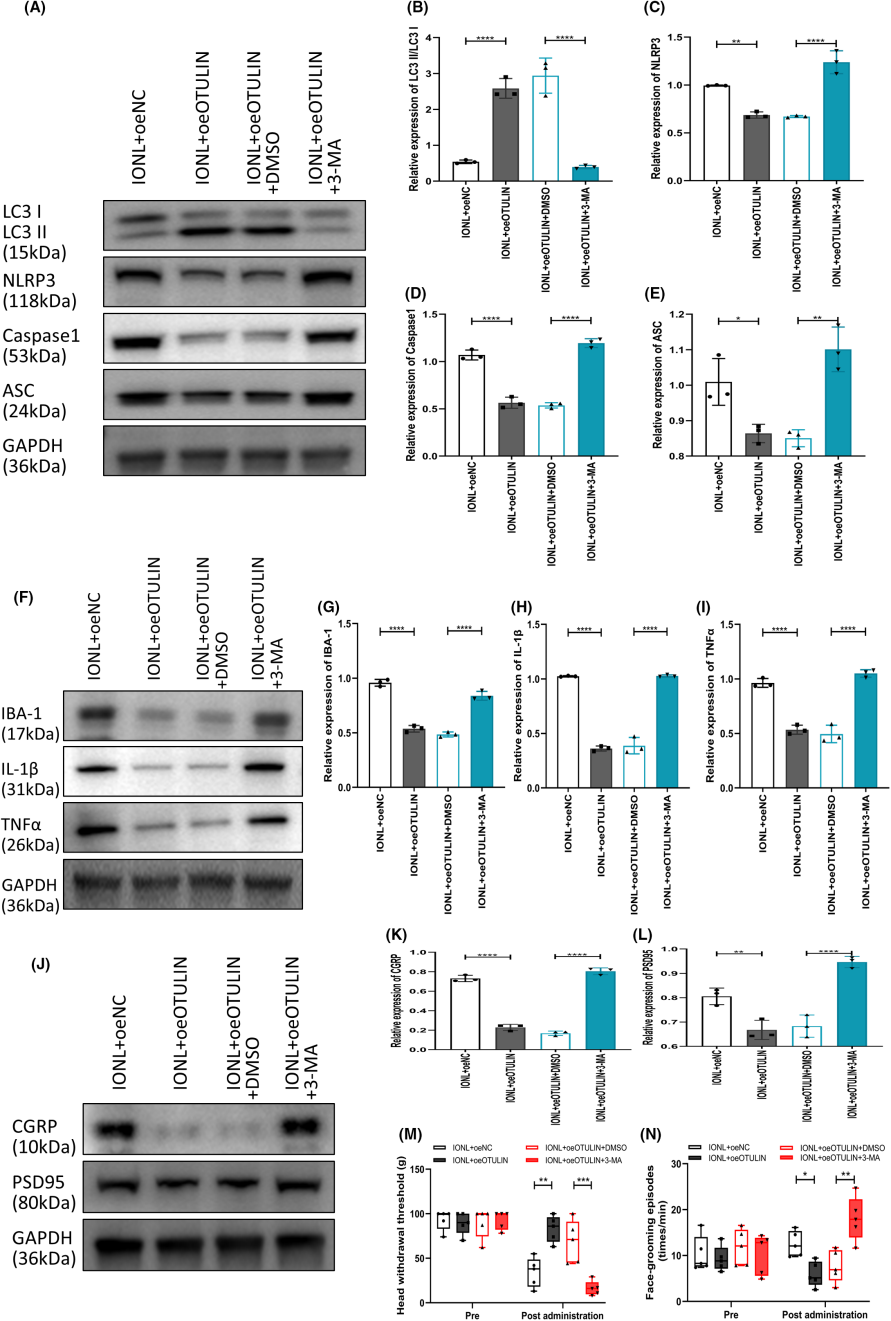
Study on the impact of OTULIN on autophagic activity and NLRP3 inflammasome in IONL‐induced trigeminal ganglia. (A, B) Immunoblotting evaluated the effect of OTULIN on the autophagy marker LC3II/LC3I ratio. (C–E) Immunoblotting assessed the impact of OTULIN on NLRP3 inflammasome components (NLRP3 and caspase‐1), exploring OTULIN's regulatory mechanism on NLRP3 inflammasome activity. (F–I) Further immunoblotting evaluated the expression of neuroinflammation markers (IBA‐1, IL‐1β, and TNFα), reflecting OTULIN's influence on IONL‐induced neuroinflammatory response. (J–L) Immunoblotting analyzed the expression of neuropathic pain sensitivity markers (CGRP and PSD95), assessing OTULIN's regulatory effect on neuropathic pain sensitivity. (M) The von Frey test assessed head withdrawal thresholds to quantify pain sensitivity. (N) The face‐grooming experiment recorded the number of face‐grooming incidents, serving as an indicator of spontaneous pain behavior. The sample size for each group was *n* = 5. Statistical significance was determined using one‐way ANOVA, with significance levels marked as *p < 0.05, **p < 0.01, ***p < 0.001, ****p < 0.0001.

**FIGURE 7:**
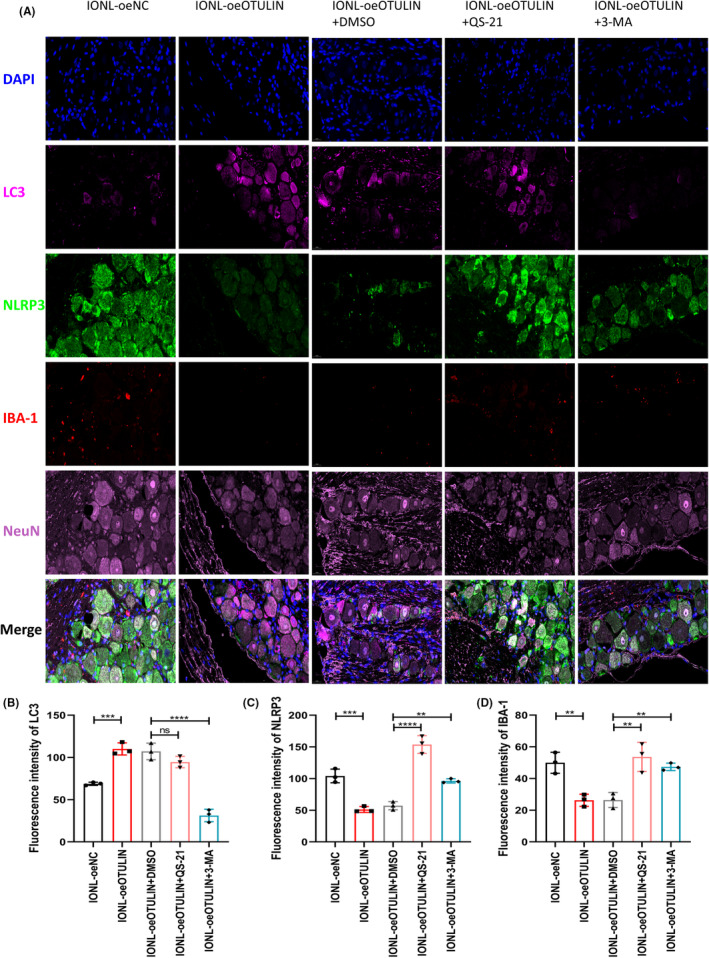
Relationship between OTULIN activation of autophagy and expression of NLRP3 inflammasome and neuroinflammation markers. (A) Immunofluorescence observed the expression of LC3, NLRP3, and IBA‐1 in IONL‐TG models post‐OTULIN upregulation, using fluorescent colors to differentiate and mark various proteins and their cellular localization and expression levels. Nuclei were stained blue with DAPI; LC3 protein was marked with purple fluorescence; NLRP3 inflammasome component expression was marked with green fluorescence; IBA‐1, indicating microglial activation, might be marked with red fluorescence; NeuN was marked with deep purple fluorescence to identify neurons. (B) LC3II fluorescence intensity was detected with specific antibodies for quantitative analysis of autophagic activity. (C) Quantitative analysis of NLRP3 fluorescence intensity, assessing the impact of OTULIN on NLRP3 inflammasome activity. (D) Quantitative analysis of microglial marker IBA‐1 fluorescence intensity, evaluating OTULIN's regulatory effect on neuroinflammatory status. The sample size for each group was *n* = 5. Statistical significance was determined using one‐way ANOVA, with significance levels marked as *p < 0.05, **p < 0.01, ***p < 0.001, ****p < 0.0001.

This study assessed the impact of OTULIN on the autophagy marker LC3II/LC3I ratio, as well as its effects on the expression levels of NLRP3 inflammasome components, neuroinflammatory markers, and neuropathic pain sensitivity markers through immunoblotting experiments. The results demonstrated that OTULIN upregulation significantly increased the LC3II/LC3I ratio (Figure 6A,B) while inhibiting the NLRP3 inflammasome (Figure 6A,C–E), neuroinflammation (Figure 6F–I), and neuropathic pain sensitivity (Figure 6J–N). The reversibility of this process was validated using the autophagy inhibitor 3‐MA, which was able to reverse the changes induced by OTULIN. Moreover, the combination of oeOTULIN and 3‐MA reversed the inhibitory effects of OTULIN on NLRP3 and IBA‐1, as well as the activation effect on LC3II (Figure 7A–D).

This study reveals the mechanism by which OTULIN inhibits the NLRP3 inflammasome in IONL‐TG through autophagy activation, offering new insights into the regulatory mechanisms of neuropathic pain. Furthermore, it provides a scientific basis for the development of novel pain treatment strategies based on autophagy regulation.

### 3.7 OTULIN suppresses LPS‐induced inflammation in microglia and neurons by modulating inflammatory responses

Further investigation focused on the role and mechanism of OTULIN protein in regulating inflammation within a lipopolysaccharide (LPS)‐induced inflammation model of rat TG primary cultured microglia (MG) and neurons.

**FIGURE 8:**
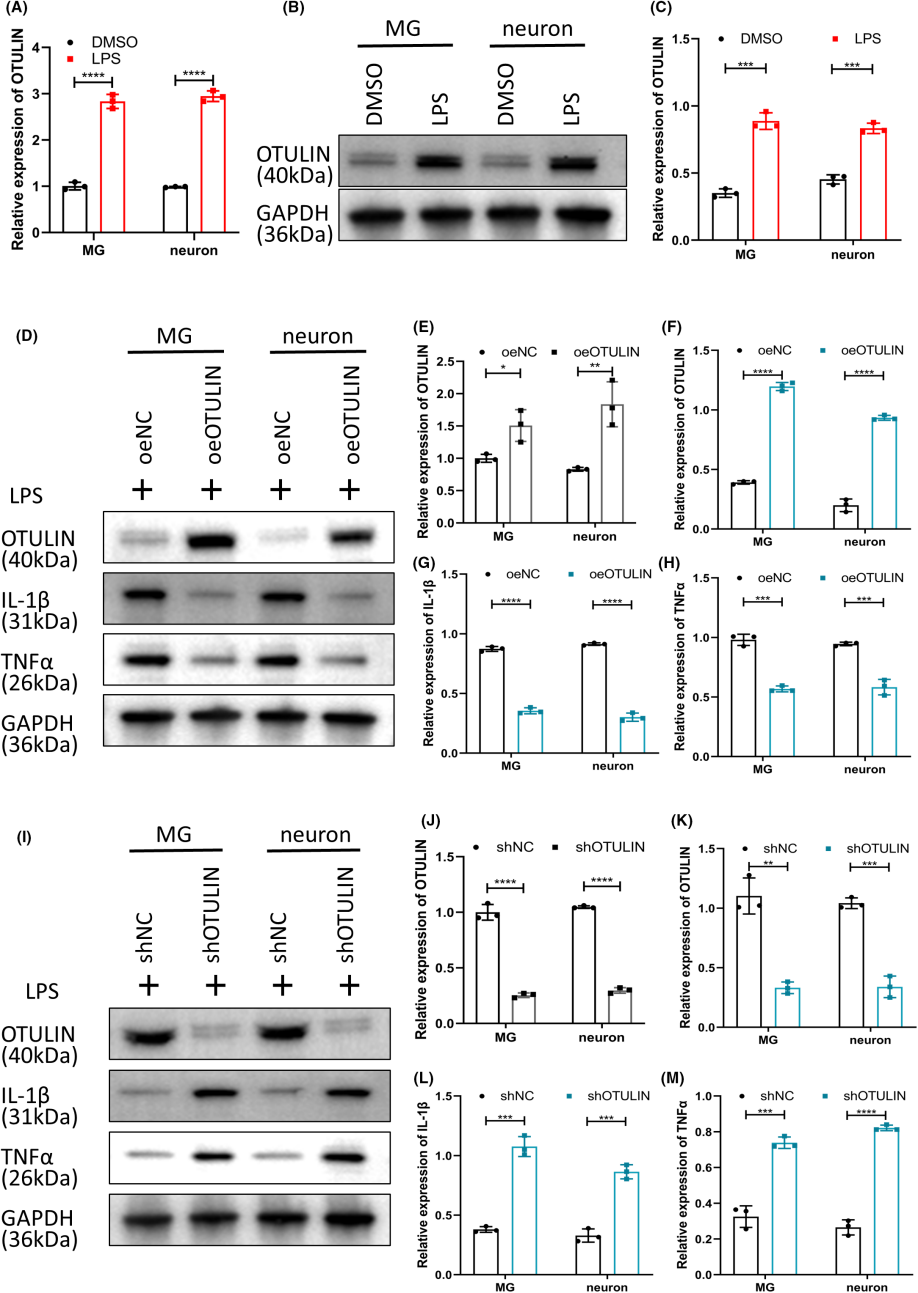
Regulation of OTULIN on LPS‐induced inflammation in microglia and neurons in vitro. (A) Real‐time quantitative PCR (qRT‐PCR) analysis assessed the impact of lipopolysaccharide (LPS) treatment on OTULIN mRNA expression in microglia and neurons. (B) Immunoblot analysis of the relative expression levels of OTULIN protein in microglia and neurons post LPS treatment. (C) qRT‐PCR analysis of the relative expression levels of OTULIN mRNA in microglia and neurons post LPS treatment. (D, I) Immunoblotting verified the transfection efficiency of OTULIN overexpression and silencing vectors. (E, J) qRT‐PCR experiments evaluated the expression of OTULIN mRNA in microglia and neurons post‐vector transfection. (F–H, K–M) Immunoblotting assessed the regulatory effect of OTULIN expression on the expression of inflammation markers IL‐1β and TNFα in microglia and neurons. The sample size for each group was *n* = 5. Statistical significance was determined using one‐way ANOVA, with significance levels marked as *p < 0.05, **p < 0.01, ***p < 0.001, ****p < 0.0001.

Experimental outcomes, as revealed by qRT‐PCR (Figure 8A) and immunoblotting (Figure 8B,C), indicated a significant induction of OTULIN expression in microglia and neurons following LPS treatment, suggesting a critical role for OTULIN in inflammatory responses. To further validate OTULIN's anti‐inflammatory function in vitro, specific vectors were employed to overexpress and silence OTULIN in microglia and neurons, with transfection efficacy confirmed by immunoblotting (Figure 8D,I) and qRT‐PCR (Figure 8E,J). In microglia and neurons with OTULIN overexpression, there was a significant reduction in the expression levels of inflammatory markers IL‐1β and TNFα (Figure 8D,F–H), whereas OTULIN knockdown led to an increase in these inflammatory markers (Figure 8I,K–M), further corroborating OTULIN's role in suppressing inflammatory responses.

In summary, this study elucidates OTULIN's significant inhibitory effect on LPS‐induced inflammation in microglia and neurons in vitro. OTULIN modulates the expression of inflammatory markers, playing a crucial role in the negative regulation of inflammatory responses.

### 3.8 OTULIN suppresses inflammatory marker expression in microglia and neurons by activating autophagy

In an in vitro experimental model, we explored the induction of autophagic activity by OTULIN in microglia and neurons and examined its impact on the expression of NLRP3 and IBA‐1.

**FIGURE 9:**
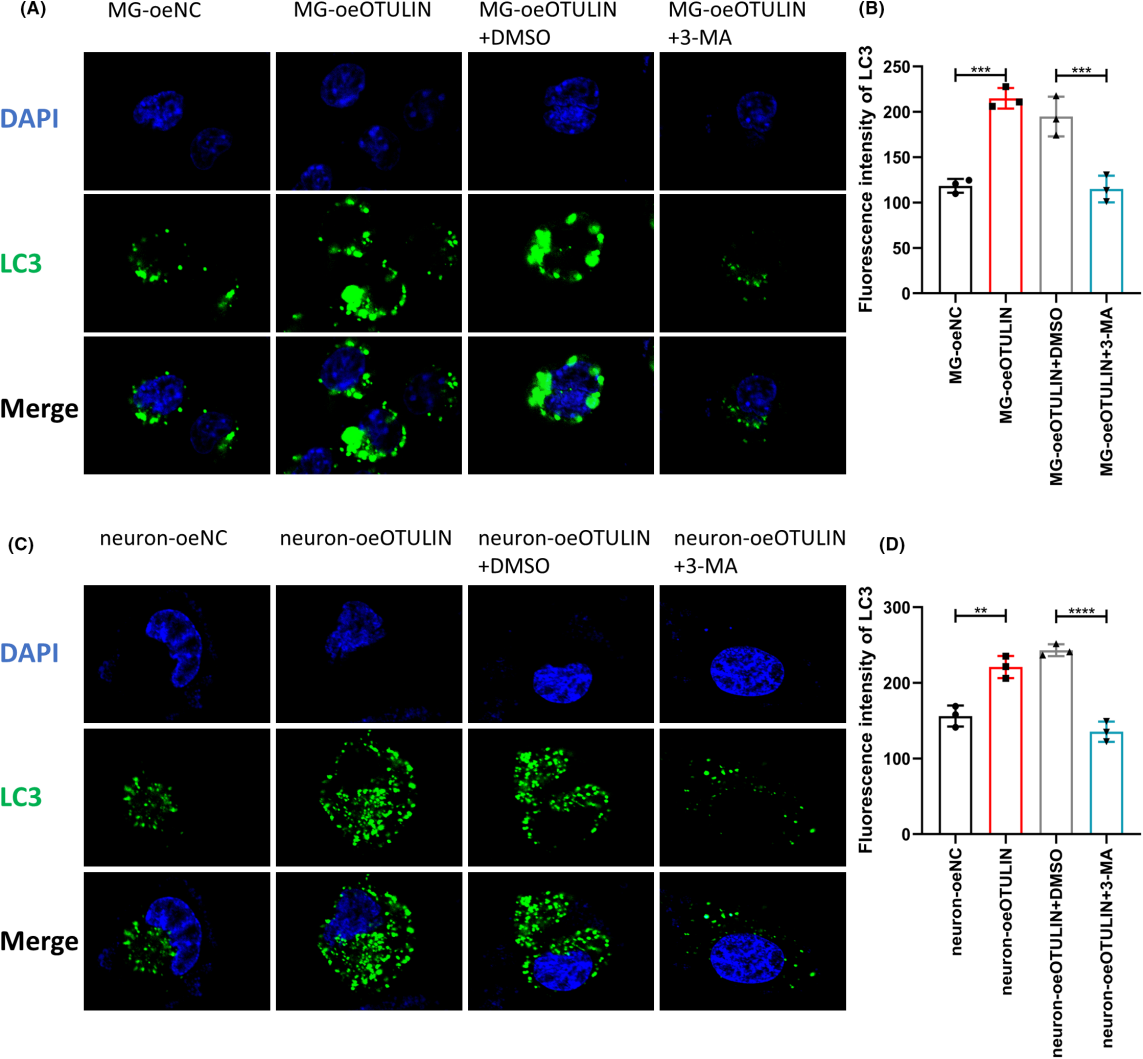
OTULIN‐induced autophagic activity in microglia and neurons. (A, B) Immunofluorescence showed the impact of OTULIN overexpression on LC3II expression in microglia. (C, D) Immunofluorescence methods displayed the effect of OTULIN overexpression on LC3II expression in neurons. The experiment also introduced the autophagy inhibitor 3‐MA to explore its impact on OTULIN‐induced autophagic activity, which is applied in both microglia and neurons. Immunofluorescence analysis used specific antibodies against LC3II, visually displaying the formation and distribution of autophagosomes with green fluorescence. Nuclei were stained blue with DAPI to provide background information on cell localization and morphology. The sample size for each group was *n* = 5. Statistical significance was determined using one‐way ANOVA, with significance levels marked as *p < 0.05, **p < 0.01, ***p < 0.001, ****p < 0.0001.

**FIGURE 10:**
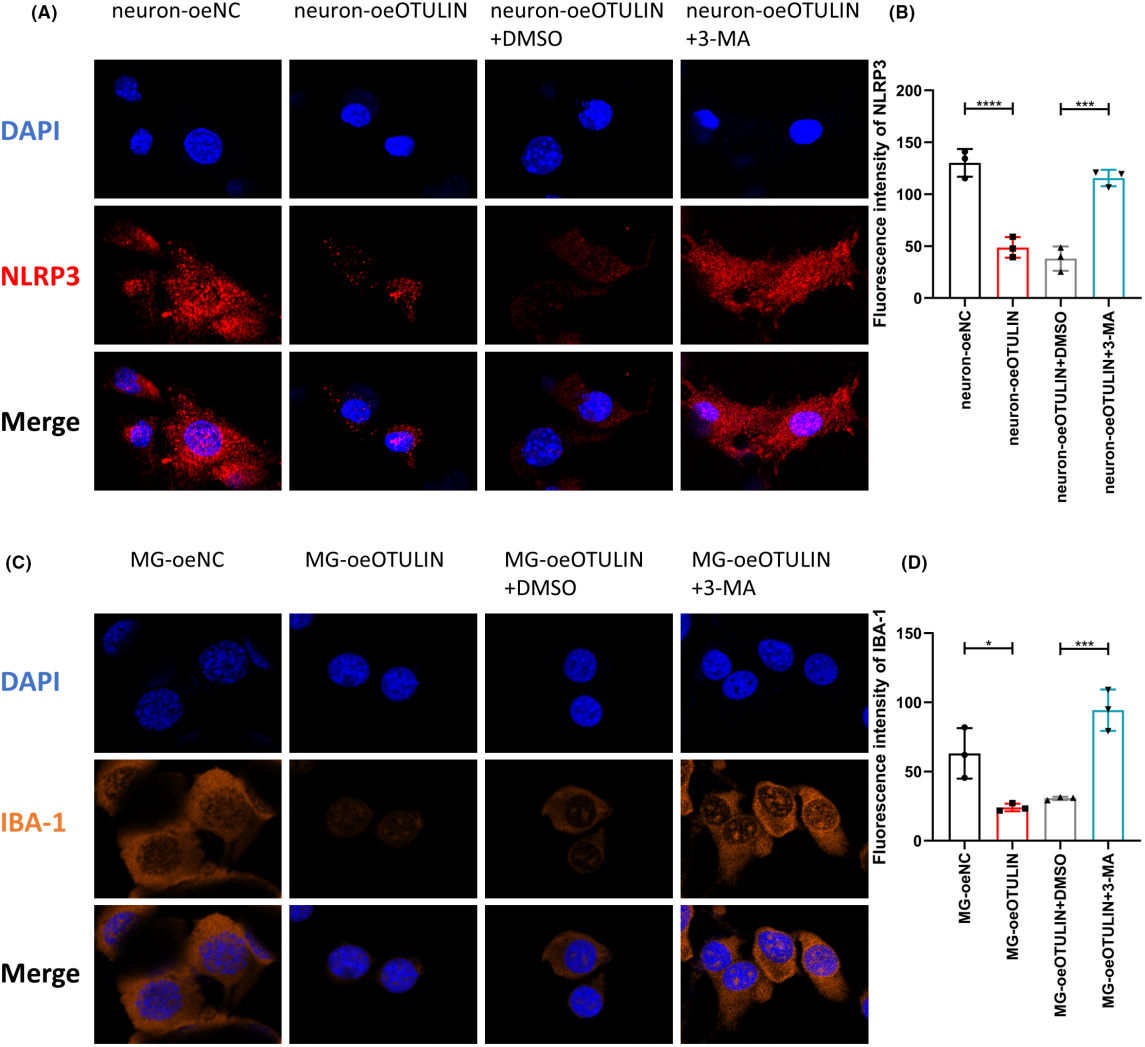
OTULIN suppresses expression of inflammatory markers in microglia and neurons through autophagy process. (A, B) Immunofluorescence methods observed the impact of OTULIN overexpression on NLRP3 expression in neurons and analyzed its fluorescence intensity. Nuclei were stained blue with DAPI, and NLRP3 was marked with red fluorescence, visually displaying the expression and distribution of inflammasome components; the reduction in red fluorescence intensity reflects a decrease in NLRP3 expression, indicating suppression of inflammatory response. (C, D) Immunofluorescence studied OTULIN overexpression's regulatory effect on IBA‐1 expression in microglia and analyzed its fluorescence intensity. Nuclei were also stained blue with DAPI, and IBA‐1, a marker of microglial activation, was marked with orange fluorescence; a decrease in orange fluorescence intensity indicates reduced microglial inflammatory activity. The sample size for each group was *n* = 5. Statistical significance was determined using one‐way ANOVA, with significance levels marked as *p < 0.05, **p < 0.01, ***p < 0.001, ****p < 0.0001.

Immunofluorescence analysis revealed that OTULIN significantly increased the expression of LC3II in both microglia and neurons (Figure 9A–D), indicating the activation of autophagy. However, the upregulation of LC3II expression induced by OTULIN was reversed upon application of the autophagy inhibitor 3‐MA (Figure 9A–D), confirming the dependency of OTULIN's function on autophagy activation. Furthermore, our immunofluorescence analysis showed that OTULIN also reduced the expression of NLRP3 in neurons and IBA‐1 in microglia (Figure 10A–D), unveiling a potential mechanism for its role in suppressing inflammatory responses. When cells were treated with both OTULIN and 3‐MA, the inhibitory effects on NLRP3 and IBA‐1 expression were reversed (Figure 10A–D), further substantiating that OTULIN suppresses the expression of inflammatory markers through the autophagy pathway.

## 4 DISCUSSION

Neuropathic pain significantly impairs patient quality of life, with TN being a critical component of chronic neuropathic pain disorders. Despite ongoing research, the pathogenesis of TN remains to be fully elucidated.^4^, ^57^, ^58^ This study delved into OTULIN's regulatory role in TN and neuroinflammation induced by IONL, particularly its mechanism of suppressing NLRP3 inflammasome activity through autophagy activation. Contrasting with previous studies, our findings unveil a dual role of OTULIN in autophagy and inflammation regulation, offering a fresh perspective on the complex mechanisms underlying neuropathic pain. While inflammation has been established as a key player in TN development, our research further refines this mechanism, demonstrating OTULIN's capacity to inhibit NLRP3 inflammasome activity via autophagy activation, thereby presenting a novel molecular target for TN treatment.

The prevailing view attributes TN's etiology to demyelination caused by neurovascular compression or tumors. However, attributing TN solely to neural compression is inappropriate, as neurovascular compression is also common in individuals without TN symptoms, and not all TN patients exhibit typical neural compression.^59^, ^60^ Thus, investigating the underlying mechanisms of TN is essential. Studies have identified neuroinflammation as a critical mechanism in the progression of neuropathic pain.^12^, ^61^, ^62^ For instance, chronic curcumin can alleviate pain behavior by reducing the expression of IL‐1β and TNFα in the hippocampus.^63^ Our study indicates significant microglial activation and upregulation of neuroinflammatory markers IBA‐1, IL‐1β, and TNFα in TGs treated with IONL and microglia/neuron cells treated with LPS. Suppressing microglial activation and the expression of IL‐1β and TNFα significantly alleviates TN symptoms.

Abnormal OTULIN function can lead to systemic inflammation in humans and mice.^64^ Specific OTULIN deficiency can cause chronic liver inflammation and cancer in mice.^65^, ^66^ Moreover, OTULIN overexpression has been reported to inhibit the NF‐κB pathway in a rat model of cerebral ischemia/reperfusion, improving microglial activation and neuroinflammation.^40^ TNFs play a crucial role in OTULIN deficiency‐related inflammation, with anti‐TNF antibodies effectively treating inflammation induced by OTULIN deficiency. Our study is the first to report OTULIN's role in neuropathic pain, showing protective effects in TN neuropathic pain and neuroinflammation, similar to previous studies. OTULIN overexpression suppressed the expression of neuropathic pain markers CGRP and PSD95, and neuroinflammatory markers IBA‐1, IL‐1β, and TNFα were all downregulated.

Growing evidence suggests that excessive activation of the NLRP3 inflammasome is associated with various inflammatory diseases.^67^, ^68^, ^69^ NLRP3 activation triggers the maturation and secretion of IL‐1β and IL‐18, leading to a cascade of inflammatory responses in the nervous system.^25^ Animal model studies have found that inhibiting NLRP3/IL‐1β signaling can effectively alleviate neuralgia.^70^ Mechanistically, IL‐1β produced by the NLRP3 inflammasome can mediate neuropathic pain through pathways such as JNK, P38/MAPK, JAK/STAT3, and GRK2.^71^, ^72^

Autophagy, a critical metabolic pathway, plays an essential role in maintaining cellular equilibrium, manifesting in two forms: non‐selective and selective autophagy. Non‐selective autophagy allows cells to recycle nutrients under energy‐limited conditions, whereas selective autophagy facilitates the removal of intracellular organelles to preserve cellular structure.^73^ Previous research presents inconsistent findings; autophagy can both inhibit and promote the NLRP3 inflammasome under certain conditions.^74^ The significance of autophagy in neuroinflammation has been recognized,^75^ acting as a major regulator of the NLRP3 inflammasome by eliminating inflammasome components, suppressing excessive inflammation, and inhibiting the activation of the NLRP3 inflammasome and subsequent neuropathic pain signaling.^76^ When NLRP3 inflammasome is stimulated in monocytes, it binds to autophagosomes and is subsequently engulfed and degraded by them.^74^ Studies show that the lack of the autophagy‐related protein ATG16L1 in mouse embryonic liver‐derived macrophages leads to caspase‐1 activation and IL‐1β secretion.^77^ Similarly, macrophages with downregulated ATG7 or treated with the autophagy inhibitor 3‐MA show markedly enhanced IL‐1β secretion.^77^ Conversely, autophagy can also promote NLRP3 inflammasome activation in yeast, activating caspase‐1 through a non‐classical pathway during cellular starvation, increasing IL‐1β synthesis, and exacerbating tissue inflammation.^78^ However, this function seems unique to yeast. Our study revealed a significant reduction in autophagy activity in TN and demonstrated that OTULIN can induce autophagy activity, thereby inhibiting the NLRP3 inflammasome and alleviating neuroinflammation and neuropathic pain.

Regarding the potential clinical application of OTULIN, our findings support the concept of OTULIN as a new target for treating TN. The strategy of inhibiting NLRP3 inflammasome activity by activating the autophagy pathway provides a scientific basis for developing new treatment methods. However, translating these findings into clinical applications requires further research, especially in verifying the effects and safety of OTULIN in human patients.

Lastly, we must acknowledge the limitations of this study, including biases that may arise from the use of specific animal models and experimental methods. Future research should aim to validate and expand our findings through a broader range of models and techniques, particularly exploring OTULIN's role in human TN patients. Despite these limitations, our study clarifies OTULIN's function in alleviating neuropathic pain and neuroinflammation by activating the autophagy pathway, offering valuable insights into the molecular mechanisms of TN, and developing new therapeutic strategies.

By delving into OTULIN's role in TN, this study not only enhances our understanding of the mechanisms behind neuropathic pain but also provides new directions for future clinical research and therapeutic strategy development. As our comprehension of these complex mechanisms deepens, developing more effective treatment methods becomes possible, ultimately improving the quality of life for TN patients.

## 5 CONCLUSION

In summary, OTULIN significantly mitigates neuropathic pain and neuroinflammation in the IONL rat model by activating the autophagy pathway and inhibiting the activity of the NLRP3 inflammasome. These findings highlight OTULIN's pivotal role in modulating inflammatory signaling pathways and autophagic processes, providing new theoretical foundations and molecular targets for the treatment of TN and other neuroinflammatory diseases.

This study unveils the crucial function of OTULIN protein in regulating the IONL‐induced rat model of TN, particularly its capacity to alleviate neuropathic pain and neuroinflammation through the activation of the autophagy pathway and suppression of the NLRP3 inflammasome activity. This discovery not only offers a fresh perspective on the pathogenesis of neuropathic pain but also identifies OTULIN as a potential target for future treatments of TN and related inflammatory neurological disorders, underscoring its significant value in scientific research and clinical applications.

## AUTHOR CONTRIBUTIONS

Q.D. and W.H.Y. are responsible for the design of the study; H.Y.W. is responsible for experimental implementation; H.W. is responsible for the collection and analysis of data; W.H.Z. is responsible for writing the manuscript; D.W. and C.L.S. are responsible for reviewing the manuscript. Jun Dong and all authors contributed to the revision and approved the manuscript.

## FUNDING INFORMATION

This study was supported by grants from Zhejiang Provincial Natural Science Foundation of China (grant no. LHZY24H090004), the agricultural and social development project of Hangzhou (20211231Y025; 202204B11), Zhejiang Medical and Health Science and Technology Program (2024KY1327; 2023KY950), and Zhejiang Provincial Health Technology Plan (grant no. 2022KY945; grant no. 2021KY229).

## CONFLICT OF INTEREST STATEMENT

The author declares no conflict of interest.

## CONSENT

Not applicable.

## Supporting information


Figure S1.

## Data Availability

The data that support the findings of this study are available on request from the corresponding author upon reasonable request.
