# Supplementary figures and images for: OTULIN's influence on neuroinflammation and pain modulation in trigeminal neuralgia
Source: CNS Neurosci Ther. 2024 Aug 22;30(8):e70006. doi: 10.1111/cns.70006 (PMC11339468; doi:10.1111/cns.70006)

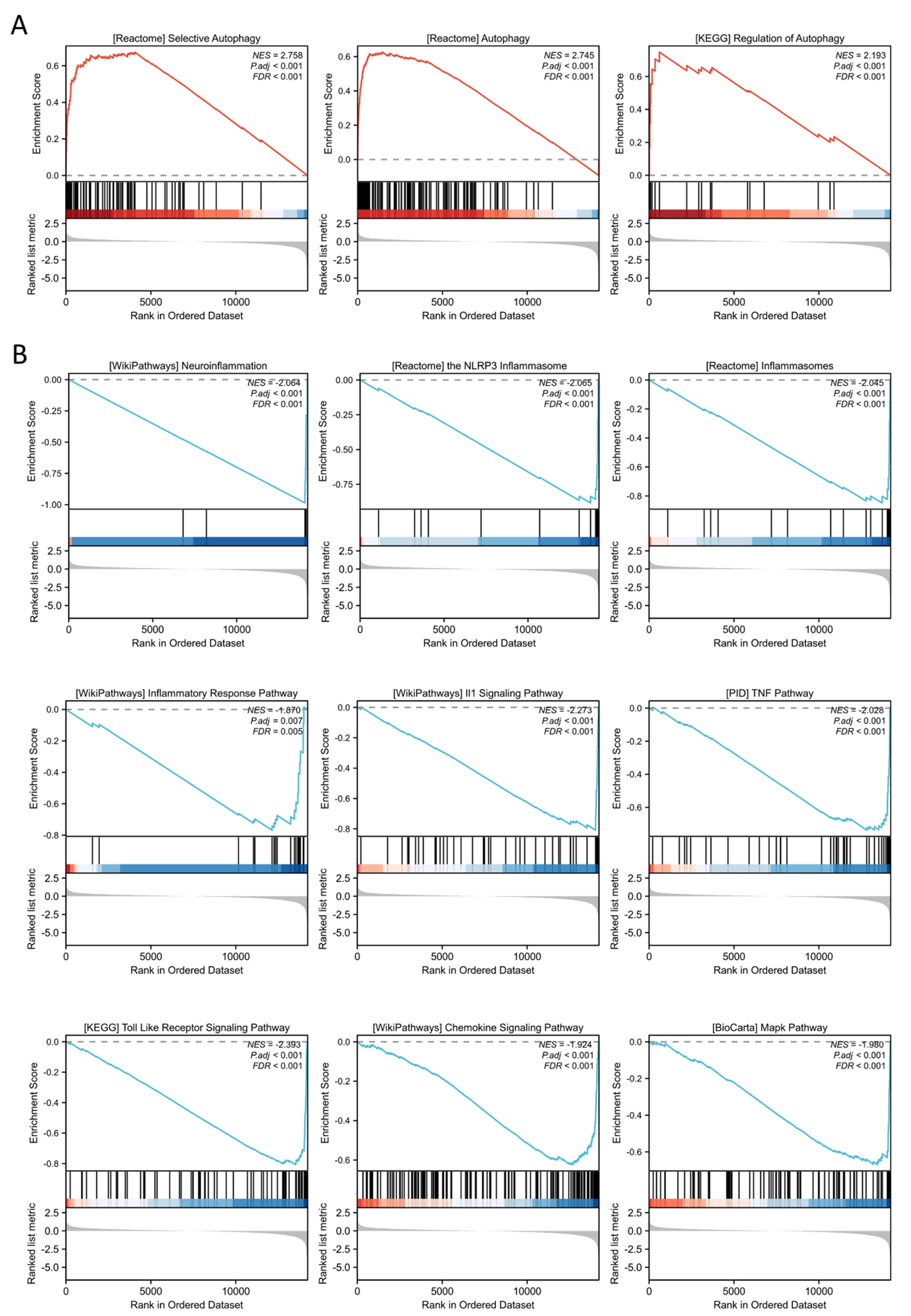

Supplement: Supplementary file 1 — Figure S1. [file CNS-30-e70006-s001.jpg]
